# Experience of using video support by prehospital emergency care physician in ambulance care - an interview study with prehospital emergency nurses in Sweden

**DOI:** 10.1186/s12873-021-00435-1

**Published:** 2021-04-07

**Authors:** Veronica Vicente, Anders Johansson, Magnus Selling, Johnny Johansson, Sebastian Möller, Lizbet Todorova

**Affiliations:** 1 The Ambulance Medical Service in Stockholm (AISAB), Lindetorpsvägen 11, SE-121 18 Johanneshov, Stockholm, Sweden; 2 Academic EMS, Stockholm, Sweden; 3 Karolinska Institute, Department of Clinical Science and Education, Södersjukhuset in Stockholm, Stockholm, Sweden; 4 Office of Medical Services, Region Skåne, Malmö, Sweden; 5 Department of Clinical Science, Lund University, Region Skåne, Lund, Sweden

**Keywords:** Prehospital care, Ambulance nurse, Video consultation, Physician participation, Teamwork, Patient safety

## Abstract

**Introduction:**

When in need of emergency care and ambulance services, the ambulance nurse is often the first point of contact for the patient with healthcare. This role requires comprehensive knowledge of the ambulance nurse to be able to assign the right level of care and, if necessary, to provide self-care advice for patients with no further conveyance to hospital. Recently, an application was developed for transmitting real-time video to facilitate consultation between ambulance nurses and prehospital physicians in the role of regional medical support (RMS) for ambulance care. The use of video communication as a complement of medical support when referring to self-care is still an unexplored method in a prehospital setting.

Our study aimed to elucidate ambulance nurses’ experience of video consultation with RMS physician during the assessment of patients considered to be triaged to self-care.

**Method:**

We conducted a qualitative design study using semi-structured interviews with open questions. Twelve ambulance nurses were included in the study. To explore the ambulance nurses’ experience of performing video consultation with RMS physician, in cases when a patient was assessed and triaged to self-care, a content analysis was performed.

**Results:**

A main category emerged from the results: “ Video consultation as decision support in the ambulance care promotes increased patient participation and for the ambulance nurses, it creates a feeling of increased patient safety “. The main category was based and formed on the following categories: “ Simultaneous presence of ambulance nurse and a physician increases patient participation during the assessment resulting in a confident care decision “. “Interprofessional collaboration strengthens the medical assessment”. “Video technology promotes accessibility for patients needs in the ambulance care regardless of emergency level”.

**Conclusions:**

Ambulance nurses experienced that the use of video consultation increases patient involvement and confidence in healthcare when both the ambulance nurse and the physician were present when deciding on self-care advice. The live imaging allowed the ambulance nurse and prehospital physician to reach a consensus on the patient’s current medical care needs, which in turn led to a feeling of increased patient safety for the ambulance nurses.

## Introduction

Worldwide, approximately 50% of the ambulance patients are classified as non-urgent by the dispatcher, according to emergency medical services (EMS) data [1, 2]. Prehospital emergency nurses and paramedics’ assessment of ambulance patients indicates that only about 70% actually require conveyance to an ED [3–5]. Thus, almost one-third of the non-urgent ambulance patients could be referred to alternative care levels, such as primary healthcare units [6, 7] or left at the scene with self-care clinical advice [8]*.* In a prehospital setting, it is not always simple to determine the right medical treatment and provide the right level of healthcare. A recent study has shown that in one-third of the emergency calls that were attended by ambulance but not transported to a hospital, the majority of patients did not have a subsequent event within 3 or 7 days but a small proportion of patients did have contacts with multiple emergencies and urgent care health services within 3 days of the non-transport decision [8].

Incorrect assessments can increase patients’ suffering but in worst cases even lead to death [9–11]. Assessment and triaging patients to self-care without conveyance to the hospital puts a high level of responsibility on ambulance nurses and paramedics [12–14]. Sometimes non-transport occurs due to patient refusal. According to a qualitative interview study regarding ambulance nurses experiences of assessing non-conveyed patients in the EMS by Lederman et al. (2019), ambulance nurses experienced both uncertainties regarding the accuracy of their assessment of the patients’ medical need as well as a feeling of loneliness in their decision making [15]. Although the EMS health policy promotes alternative care levels, such as non-conveyance, as means to offer care closer to patients’ homes [16], there is inadequate organizational support in the Swedish EMS for ambulance nurses’ assessments in general [15]. Better decision support systems are necessary for EMS as part of public health care, since both medical demands and technical progress are increasing worldwide. Telemedicine of the third generation is considered a safe and technically feasible solution for real-time bidirectional audio-video communication between healthcare providers and remote patients. Telemedicine in a prehospital setting has been proposed to have a positive impact on emergency medical care, to improve the pre-hospital diagnosis of stroke, myocardial infarction, and trauma [17]. A survey conducted in 2017 regarding paramedic’s perspective on prehospital telemedicine implies a potential to improve the patient care experience, including prehospital diagnosis, destination decisions, and patient satisfaction. A second opinion was valuable especially in cases where the patient diagnosis or disposition was unclear [18]. There are still few studies that have demonstrated whether it influenced clinical outcomes for patients.

Since 2009, in Sweden, there is regional medical support (RMS) telephone support around the clock available for ambulance nurses for medical advice in various situations, like triage and support in difficult or complicated situations, direct prescription of medicines, and/ or consultations regarding patients who are referred to care levels other than the hospital. The RMS physician should always be contacted when a patient in need of emergency care refuses conveyance to hospital or when a decision is made to either assign the patient to other care levels or when the patient is considered to remain at home with self-care clinical advice and instructions. In Sweden with around one million ambulance attendances annually, there is also an increasing number of ambulance assessments of non-urgent cases which compromises ambulance resources. Recently, as part of a larger development project in Sweden, [19, 20], RMS telephone support was supplemented with additional video support allowing video images to be transmitted from the patient’s bedside. In the County of Stockholm and Skåne were this study was performed, approximately 220.000 and 170,000 ambulance assessments respectively are performed annually. Around one third of the patents are after assessment left at the scene or at home with advice on self-care. Some of them are further triaged to primary care. Briefly, the ambulance nurse contacted the RMS via a video connection (instead of telephone line alone) enabling “face-to-face” communication. In order to increase the understanding of the advantages and disadvantages of mobile prehospital telemedicine, the purpose of this study was to explore the ambulance nurses’ experience of using a video application as complement support on bedside assessment and triage of patients non-conveyed to hospital and without the urgent need for emergency care.

## Methods

### Design and setting

We used a qualitative approach [21] to obtain an in-depth understanding of the ambulance nurses’ experiences when using video consultations with an RMS physician, following Consolidated criteria for reporting qualitative research, (COREQ) [22]. Data were collected using semi-structured interviews that were analyzed inductively using content analysis methods as described by Elo and Kyngäs [23]. The strength of the qualitative research interview is to capture and create an understanding of the informant’s approach and to try to understand their experiences from the informant’s point of view.

Municipal regulations in the two Swedish regions, Stockholm and Skåne, in which the study was conducted, require ambulances to be staffed by at least one ambulance nurse and one emergency medical technician. The study included ambulance nurses who had participated in the development project [19] and had been using video consultation support with a physician when referring patients to self-care. The request for participation in the study was sent out by e-mail to the potential informants.

The study was approved by the Regional Ethical Board in Stockholm, Sweden (Dnr 2017/2547-31/5, 2018). The informants were informed that their responses would be kept confidential and that they could withdraw from the study at any time with no explanation.

All methods were carried out in accordance with relevant guidelines and regulations.

### Data collection

Information about the project was sent by personal e-mail to all ambulance personnel in the areas where the study should take place and included a request for participation in the project. The informants were then included consecutively as they responded. Data collection was made by authors with interview competency (VV, AJ, LT), and took place after written and verbal informed consent had been obtained. The authors did not have any relationship with the informants.

Semi-structured interviews were conducted individually. Each interview was digitally recorded and started with a short presentation of the study’s aim. The interview started with an opening question: *“Can you talk to me about your experience of video consultations with the RMS function for patients to be triaged to self-care?”* The interviews then continued with follow-up questions, such as “*Can you tell me more?”* or *“What did you feel?”* based on what the informants said. After 10 interviews, a feeling of saturation arose in the material. This meant that no new information was received. To ensure that the saturation had occurred, two more interviews were conducted. All 12 interviews were performed individually and took between 16 and 28 min. The data collection took place between October and December 2018.

### Data analysis

**Fig. 1:**
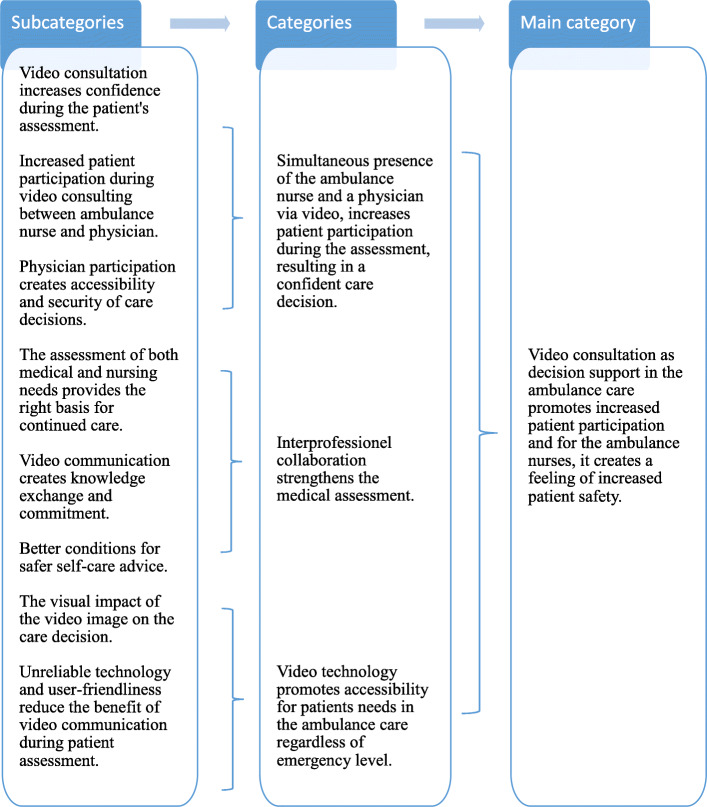
Main category, categories and subcategories based on the ambulance nurses experiences of RMS physician support via video consultation in the ambulance care when assessing non-urgent and non-convenient patients

Data were analyzed using conventional content analysis, using an inductive approach according to Elo & Kyngäs [23], which is based on three phases: the preparation phase, the organizing phase, and the reporting phase. During the preparation phase, the interviews were transcribed verbatim in their entirety and read through several times by three authors [VV, MS, JJ] to obtain a proper understanding of the content. During the organizational phase, meaningful units that corresponded to the purpose of the study were copied from the text. Each meaningful entity received a code that highlighted its content and to ensure the rigour of the data collection the interviews were compared with the transcribed text, this in a manner to verify that the interviews matched the text. The code words were then sorted into eight subcategories based on differences and similarities. The coding was made independently between the authors and then adjustments were made until consensus was reached. During the reporting phase, the results of the study are presented through systematic reporting—subcategories, categories; and the main category as presented in Fig. 1. Quotes from the interviews were used to underpin and reinforce the result. An experienced medical writer translated from Swedish to English the quotations to ensure that nothing of importance was omitted or misinterpreted.

## Results

A total of 12 ambulance nurses were interviewed, *n =* 5 (42%) were female and *n* = 7 (58%) male. All the participants had 8 ± 6 years of working experience in the EMS. From their interviews, the main category emerged: “ Video consultation as decision support in the ambulance care promotes increased patient participation and for the ambulance nurses, it creates a feeling of increased patient safety”*. This main category was based and supported by three categories;* “Simultaneous presence of ambulance nurse and a physician increases patient participation during the assessment resulting in a confident care decision”; “Interprofessional collaboration strengthens the medical assessment regardless of whether self-care was prescribed or whether the patient was transported to the hospital” and “Video technology promotes accessibility for patients needs in the ambulance care regardless of emergency level.” These categories were in turn based on eight subcategories (Fig. 1).

### Simultaneous presence of ambulance nurse and a physician increases patient participation during the assessment resulting in a confident care decision

The informants felt that video consultation with RMS increased the nurse’s and patient’s confidence in the level of care, as the physicians’ involvement created security for both. According to the informant’s experiences, this meant that the patient could receive the right care through increased involvement and that the patient’s trust and security increased when the physician participated in the communication. This category is supported by three subcategories: “Confidence in healthcare”, “Creating participation”, and “Medical inclusion creates security”.

#### Video consultation increases confidence during the patient’s assessment

The informants experienced that the video consultation gave the patients increased confidence in the care they received. They also experienced that the physician’s calm presence during the video consultation increased patient confidence in the decisions that were made, mainly because the medical assessment was more easily understood by the patient. “On one occasion they were very positive about the fact that a physician was to look at the injury”.

The analysis also revealed that the informants experienced the video consultation as increasing the duration of ambulance nurse and RMS contact with the patient. The informants experienced that this extended patient contact increased the patient’s confidence in the provided care. “It is possible that it would take a bit more time; with that said, it might be time well spent for the patient”.

#### Increased patient participation during video consulting between ambulance nurse and physician

The informants experienced that the participation of the patient and the physician (through the video conversation) increased the patient’s involvement in their care. On occasions where only the ambulance nurse and the RMS were involved in the video conversation; patient involvement did not increase. When the patient, the RMS, and the ambulance nurse were present, the informants experienced an increased opportunity for deeper discussion with the patient about continued care and self-care advice. “Well, in those cases you are really part of the discussion and can involve the patient in the video call.”

#### Physician participation creates accessibility and security of care decisions

The informants experienced that the physician’s participation through the video consultation created security for the patient when the physicians with their medical competence made their own assessment of the patient’s needs. The informants also experienced that the patients had some access to the physician. Furthermore, the informants found that the patient more easily accepted what the ambulance nurse said, as the physician’s participation created a sense of security in the patient. “They are concerned before we arrive; then we come up with an assessment and tell them that we want to consult with a physician. I think that once the physician gives his opinion, they can rest in whatever decision is made”.

### Interprofessional collaboration strengthens the medical assessment

In the analysis, it emerged that the informants experienced that video communication with the RMS resulted in better decisions regarding patient care. The interprofessional collaboration also provided the conditions for safer self-care advice and safer assessments of the diagnosis. The video communication gave the informants an increased sense of consensus with the RMS. This category is supported by three subcategories. “The assessment of medical and nursing needs is the basis for continued care recommendations”, “Video communication creates knowledge exchange and commitment”, and “Video communication creates the conditions for safer self-care advice”.

#### The assessment of both medical and nursing needs provides the right basis for continued care

The informants experienced that the initial assessment of the anamnesis and vital parameters was fundamental in their decision about the continued level of care. The informants stated that a thorough assessment of the patient sometimes gave the patient an opportunity to stay at home with advice for self-care. The informants experienced that even a deviation of a vital parameter does not necessarily mean that emergency care is the right level of care and that they were supported in this assessment when the RMS could supplement it with further questions and examinations. The informants argue that in some cases video communication with physicians can improve the assessment so that the patient is referred to the right level of care. “But if the physician gets to see the patient or how their flat looks, it may support what I say; in that way, I hope that we can arrive at a correct assessment of the patient”.

Further, the informants experienced that video communication made it easier for the ambulance nurse to reach consensus with the physician. The informants meant that, for some patients, only RMS approval was needed for consensus but in other situations, they also stated that consensus was most important in the cases where the ambulance nurse was uncertain about the assessment of the patient. When the RMS participated in the decision by video consultation, the shared responsibility created confidence for the ambulance nurses. “Yes, if I find myself in a situation where I feel uncertain in my assessment, it is reassuring to have the physician’s decision to strengthen my back”.

#### Video communication creates knowledge exchange and commitment

The informants experienced an exchange of knowledge through video communication when the RMS could supervise. The RMS was also able to show how supplementary examinations were made to improve decision-making. The informants saw the sessions as a learning opportunity that would help them with similar patient cases in the future.” *Which leads, in a similar future situation, to me having better knowledge and more advanced technique for the assessment”.*

The informants experienced that the RMS was more involved in the care of the patient when video communication was used. The informants also pointed out the importance of providing the RMS with an understanding of the complexity of the ambulance work environment. *“But if they join a video consultation, they actually participate and can see how the situation looks. That means that they increase their knowledge of how we work and the challenges and complexity of prehospital care”.*

#### Better conditions for safer self-care advice

The informants experienced that the confidence in self-care counselling increased when several people with different skills were involved in the patient’s care. In some cases, the informants found it difficult to provide self-care advice, especially when the ambulance nurse felt that he or she had inadequate knowledge in the area. With video communication, the RMS, possessing higher medical competence, could help provide self-care advice. *“There are things I may miss, but we are always two pairs of eyes in the ambulance and can discuss the patient with each other; if we then include a physician, we add another layer and “flesh to the bone”. So, of course, it means increased patient safety”.*

The analysis also revealed that the informants did not always think video communication was necessary for providing self-care advice. The informants experienced the use of video communication situationally. In most patient cases, the informants felt confident in their assessment and that their knowledge of self-care was sufficient to advise the patient.” *Not that it will be used for every patient that is triaged to self-care. But for a limited group of patients, I clearly see it as a big advantage”.*

### Video technology promotes accessibility for patients needs in the ambulance care regardless of emergency level

According to informants, video communication increased the accessibility of the patient. The informants argued that video with good image quality can contribute to safer care in all parts of ambulance care. This category is supported by two subcategories: “*The visual impact of the image on the decision*” and “*Unreliable technology and cumbersome user-friendliness reduce the benefit of video communication*”.

#### The visual impact of the image on the care decision

This subcategory describes video communication as being very helpful in those occasions when the verbal explanation via telephone was difficult. The informants felt that with injured patients it was an asset to be able to visually show the injury instead of just describing it to the RMS. On occasions with deviating parameters such as increased respiratory rate, in an otherwise unaffected patient, the informants perceived that the image could enhance the ambulance nurses’ assessment for the RMS. *“When the RMS was able to see the patient it became clear that she (the patient) actually was sad and very upset about something, which made it look as though she had an underlying respiratory problem; in fact, she was upset about her experience of having difficulty breathing caused by her emotions, creating a vicious cycle”.*

The informants experienced that the picture simplified the explanation of the patient’s condition because they could now show the patient to the RMS instead of only describing the patient verbally. All informants stated that being able to show something while giving oral information gave a more accurate picture of the situation and they believed that video communication could help the RMS to visualize the problem on the occasions when the ambulance nurse was hesitant in his assessment.” *This function is particularly useful in situations that are difficult to describe in words and convey a correct view, such as wounds or injuries”.*

#### Unreliable technology and friendliness reduce the benefit of video communication during patient assessment

Some informants described the technology as complicated. There were far too many steps to be taken before the video consultation could begin. The informants thought that the recording set-up with two pieces of equipment, one for sound and the other for imaging, was difficult to handle and therefore sometimes affected user-friendliness. The informants talked about occasions with poor connectivity which made logging into the system a problem. *“But is very important that these devices and the video transmission work. If they start to malfunction or I can’t hear, or I misunderstand—I [mistakenly] push a button somewhere or I have sound issues—then you want to throw the device out of the window. It is a requirement that these transmissions work”.*

The informants believed that optimal image quality for video communication was necessary to be able to view details and reflect a meeting. The informants thought that with poor picture and sound quality, video communication did not add value, but rather became detrimental. “*Poor picture quality does not get the right view in skin texture and so on. Or show if a wound need to be assessed. Good image quality is important.”*

### Video consultation as decision support during ambulance care creates conditions for participation and increased patient safety

The results showed that the informants’ experience of video communication as decision support opened a new dimension of participation for the patient, the ambulance nurses, and for the physician in the care meeting. The interaction that arose between the parties increased participation in the decision about self-care. The informants also experienced that the patients had increased confidence in the care and that the interaction between the ambulance nurses and the physicians created security in their decision to recommend self-care for the patient. Patient safety was expected to be improved by including the physician’s medical expertise through the video consultation. Using video, the physician and the ambulance nurse could achieve consensus on the patient’s current medical care needs. The increased security in decision-making by the informants was considered a consistent strength of video consultation regardless of the situation, whether it reduced or eliminated uncertainty or just confirmed the ambulance nurse’s assessment.

## Discussion

Our results showed that the informants’ experience of video communication as decision support opened a new dimension of participation for the patient, the nurses, and the physician in the care meeting. The opportunity for interaction between the parties increased participation in the decision on self-care. The informants also felt that the patients had increased confidence in the care and that the interaction between the nurses and the physicians created a sense of security in their decision to recommend self-care for the patient.

To be able to provide good care, it is important that the ambulance nurse is present and responsive to what the patient says, while also linking the patient with the RMS physician. Sometimes patients lack confidence in healthcare and become uncertain and begin to question the care they receive if told they should not go to the hospital [24]. Our informants felt that the physician involvement through their “presence in the room” i.e. video communication, affected patient participation in a positive way. Several studies have shown that meeting by video is almost equivalent to meeting face-to-face [25, 26].

A thorough initial assessment is important in choosing the right level of care [24, 27–29]. In our study, physician participation was perceived by the ambulance nurses as creating security for the patient. This was interpreted by the informants as the patient experiencing greater safety when members of different professions were involved in their medical assessment. Our findings are in accordance with a previous study by Vicente et al. [20], where physicians were shown to experience that their involvement by video communication increased their feeling of being more satisfied through a sense of increased patient safety. Moreover, the RMS physician was able to make his own assessment of what he or she saw, in addition to what the ambulance nurse reported [20]. Barret (2017) found that in a multi-party consultation, everyone is involved as if they were in the room, and this is reinforced even more during video consultation when they can really see what is being assessed [30]. The informants in our study expressed that video communication with the physician to support the assessment led to decisions that were perceived to improve patient safety. This probably also means that more patients can be referred to care levels other than emergency care, which might in turn lead to a reduced load on the emergency rooms. However, we found that on some occasions, the ambulance nurse simply sought the approval of the physician. On those occasions, the ambulance nurse was certain of their own assessment and felt that video communication was unnecessary. We interpreted this as being related to knowledge and experience, and that video communication is a good tool in situations where the required knowledge and experience are missing.

The informants reported that the technology needed to be optimized to become more user-friendly. Not being able to have their hands free because there were two separate devices to operate was experienced as an obstacle. The informants also felt that communicating by video took more time than the traditional telephone call. Previous studies reveal that time is a key aspect of patients’ survival [31–33]. Furthermore, the time consumed for operating the equipment may impact the time opted for the patient-ambulance nurse encounter. However, in this study ambulance nurse did not see this as something negative, but felt that the extended time with the patients gave the patients confidence and security and that the patients felt that the health care service devoted more time to their care. These findings mean that when the ambulance nurse takes the time to listen to the patient describing their situation, the patient’s confidence in the health care they receive is strengthened [34]. Nevertheless, we conclude that the technology should be made more user-friendly.

Our study suggests that video consultation, as decision support in ambulance care, creates conditions for increased participation and patient safety. Similar conclusions were obtained based on the physician’s perception using video consultation [20] suggesting that the use of telemedicine increases patient safety through improved assessment. Different routines already exist for communicating with physicians by telephone to strengthen patient safety. Our result showed that the RMS physician further strengthened patient safety through his visual presence and the opportunity to explain, in a more pedagogic manner, supplementary assessments that the ambulance nurse could use for similar cases in the future. The informants said that “a picture is worth a thousand words” and that with a picture having optimal image quality as an aid, nuances and phenomena could be more easily distinguished and explained. High-quality video and audio, and related technical issues are thus of great importance. Telemedicine in prehospital setting, according to our informants, would generate safer care and promote high-quality emergency care for the patient with non–life-threatening conditions.

### Limitations of the study

A qualitative method with an inductive analytical approach is considered a strength when the goal is a deeper understanding of unique information [35]. However, it needs to be considered that it may be difficult to draw any general conclusions, because of the small number of informants. However, our study reports the ambulance nurses’ experience of video communication, which we believe together with credibility and confirmability becomes generalizable to similar contexts to the ambulance services in other regions of Sweden [36].

The interviews lasted 16–28 min, which may be considered a weakness because qualitative interviews can last for hours to thoroughly explore a phenomenon [36]. Our interviews had no definite time limit but continued until the interviewer had covered the different aspects of the ambulance nurse’s experience and the ambulance nurse felt they had no more information to offer. The 12 informants’ deep descriptions of their experiences were judged rich in content with great variance. Before the interview ended, all the participants were asked whether they had anything to add, which we consider strengthened our results.

## Conclusions

The results of our study indicate that video consultation strengthens the involvement of the patient, when both the ambulance nurse, and the RMS physician are working together with the patient to reach a decision regarding non-conveyance to hospital and self-care advice. It further shows that ambulance nurses’ interactions with the RMS also increased the ambulance nurses’ confidence in such assessment to advice self-care for the patient. By means of video, the ambulance nurses and the physician could reach a consensus on the patient’s current medical care needs, and this was felt to be accompanied by increased accessibility and credibility for the patient. It also created a sense of increased patient safety for the ambulance nurses. Still, to obtain a more complete picture of the use of video communication in ambulance care and how the patients experience the provided care, further research is required. The present study does not elucidate the patient’s perspective nor the medical outcome. It would also be of great importance to gain further knowledge about the power balances between patient and ambulance nurse and consequences of it (e.g. asymmetrical caring relationship) and how this may be affected by the presence of a physician. Nevertheless, the used video concept in prehospital setting we present here, should be further developed to ensure that minor audio and image weaknesses, and other technical issues, do not cause unnecessary delays and interruptions in the patient assessment and during the consultation between the ambulance nurse and the RMS.

## Data Availability

The data in this study are available from the corresponding author upon request.

## Abbreviations

EMS: Emergency medical services
RMS: Regional medical support
